# Comparison of percutaneous cross screw fixation versus open reduction and internal fixation for pelvic Day type II crescent fracture-dislocation: case-control study

**DOI:** 10.1186/s13018-020-02197-1

**Published:** 2021-01-09

**Authors:** Guangheng Xiang, Xiaoyu Dong, Xingan Jiang, Leyi Cai, Jianshun Wang, Xiaoshan Guo, Jian Xiao, Yongzeng Feng

**Affiliations:** 1 Department of Orthopaedic, The Second Affiliated Hospital and Yuying Children’s Hospital of Wenzhou Medical University, Wenzhou, Zhejiang, 325035 China; 2 School of Pharmaceutical Sciences, Wenzhou Medical University, Wenzhou, Zhejiang, 325035 China

**Keywords:** Day type-II crescent fracture-dislocation, Minimally invasive fixation, Cross-screw fixation, Percutaneous internal fixation

## Abstract

**Objective:**

To investigate the clinical outcomes of percutaneous cross screws internal fixation for pelvic Day type II crescent fracture-dislocation.

**Methods:**

We reviewed 66 consecutive patients undergoing surgical treatment for Day type II crescent fracture-dislocation from June 2005 to December 2017. Percutaneous cross screws internal fixation was performed in 40 patients, and open reduction and internal fixation was performed in 26 patients. The patient characteristics, surgical complications, radiographic and clinical outcomes and were compared.

**Results:**

There was no statistically difference on the mean time from injury to surgery between the two groups. The time of operation, the amount of blood loss, the length of incision, and the hospital stay were significantly shorter in the percutaneous cross screws internal fixation group. No significant difference on Matta scores and Majeed scores between the two groups. The open reduction and internal fixation group resulted in a higher rate of intraoperative hemorrhage, nerve injury, discomfort, and pain.

**Conclusion:**

Percutaneous cross screws internal fixation for Day II type pelvic crescent fracture-dislocation was safe and effective. Minimally invasive fixation had the advantages of short operation and hospitalization time, less intraoperative bleeding, and surgical trauma.

## Introduction

Pelvic fractures are often caused by high-energy injuries, often accompanied by abdominal organs, blood vessels, nerve injuries, and with many complications and high mortality [1–4]. Once the pelvic fracture affects the integrity of ligaments and muscle structures, the stability of the sacroiliac joint will also be destroyed, causing the posterior ring of the pelvis to become unstable [5].

Pelvic crescent fracture and dislocation were first reported by Borrelli et al. in 1996 [6, 7]. It was a sacroiliac joint complex injury, and the fracture of the iliac bone extended from the sacroiliac joint site upwards to the iliac crest, with a partial dislocation of the anterior sacroiliac joint. Day et al. divided crescent fracture-dislocation (CFD) into three types: type I fracture involved the anterior 1/3 of the sacroiliac joint, type II fracture involved the middle 1/3 of the sacroiliac joint, and type III fracture involved the posterior 1/3 of the sacroiliac joint [8]. For the treatment of type II fractures, it was usually carried out by anterior or posterior approach open reduction and internal fixation (ORIF) [9–11]. But there was still some controversy about the surgical trauma and internal fixation methods [12].

Since June 2005, our department has begun to treat patients with Day type II CFD using percutaneous posterior iliac screw combined with sacroiliac joint screw fixation. In previous studies, we have proved that percutaneous cross screw fixation was a reliable method for the treatment of Day type II CFD through a finite element analysis [13]. The aim in this study was to explore the clinical efficacy and complications of percutaneous cross screw internal fixation (PCSIF) in the treatment of Day type II crescent fracture-dislocation, and to evaluate its clinical application prospects.

## Materials and methods

### Patients

From June 2005 to December 2017, a total of 66 patients with Day type II CFD were selected and retrospectively analyzed according to the following criteria. According to different surgical methods, patients with Day type II CFD were divided into minimally invasive percutaneous cross screw internal fixation treatment group (group A, 40 cases) and open reduction and internal fixation treatment group (group B, 26 cases). The eligibility criteria were (1) Day type II crescent fracture-dislocation; (2) recipients of surgery including open or closed reduction internal fixation; and (3) complete follow-up and information. Exclusion criteria included (1) children pelvic fractures; (2) local or systemic infections; and (3) severe blood vessel and nerve injury. Patient characteristics such as age, sex, injury mechanisms, and fracture classification were extracted from the database. The data were analyzed anonymously and personal identifiers were completely removed. This study followed the guidelines of the “Declaration of Helsinki” and was approved by the hospital’s ethics committee. Written informed consent was obtained from all patients.

### Surgical technique

In our study, the anterior ring injury could be fixed in the same incision while the posterior ring was fixed by plate through the anterior approach. The rest of the patients who needed anterior ring surgery were fixed in the supine position, then the posterior ring was fixed in the prone position. Forty-six cases of anterior ring injury were treated with closed reduction screw fixation, 12 cases were treated with ORIF, and the remaining 8 patients were not fixed because the anterior ring fracture did not move significantly. Among them, there were 5 cases of pubic rami fracture with pubic symphysis separation. So they were fixed with percutaneous pubic symphysis screw and pubic rami screw in one stage.

#### Percutaneous cross screw internal fixation (group A)

One to two posterior iliac cross screws combined with 1 sacroiliac joint screw fixation in prone position. If the patient was accompanied by anterior ring pubic rami fracture or pubic symphysis separation, the anterior ring injury was first fixed with pubic rami screws or/and pubic symphysis screws.

After successful anesthesia, the patient was placed on the X-ray surgical bed with complete fluoroscopy. When the fracture displacement of the pelvis anterior and posterior rings was obvious, the anterior ring should be reset and fixed firstly. Then, we pushed the compressive iliac bone on the injured side outward manually or used a 5-mm Schanz nail to insert the edge of the iliac crest to assist the external rotation of the ilium for reduction. At the same time, external rotation and abduction of the hip joint could also play a role in assisting the external rotation of the injured side of the iliac bone. Once closed reduction was difficult, it made a small incision of 0.5 cm at the apex of the fracture and dislocation. A top rod was used to push the proximal dislocated part of the iliac posterior fracture to the distal end, assisted by longitudinal traction of the lower extremities. When closed reduction was completed, percutaneous screw fixation was performed. Generally, the sacroiliac joint screw fixation was performed before the posterior iliac screw fixation on the basis of correcting the vertical displacement of the posterior iliac fracture.

**Fig. 1:**
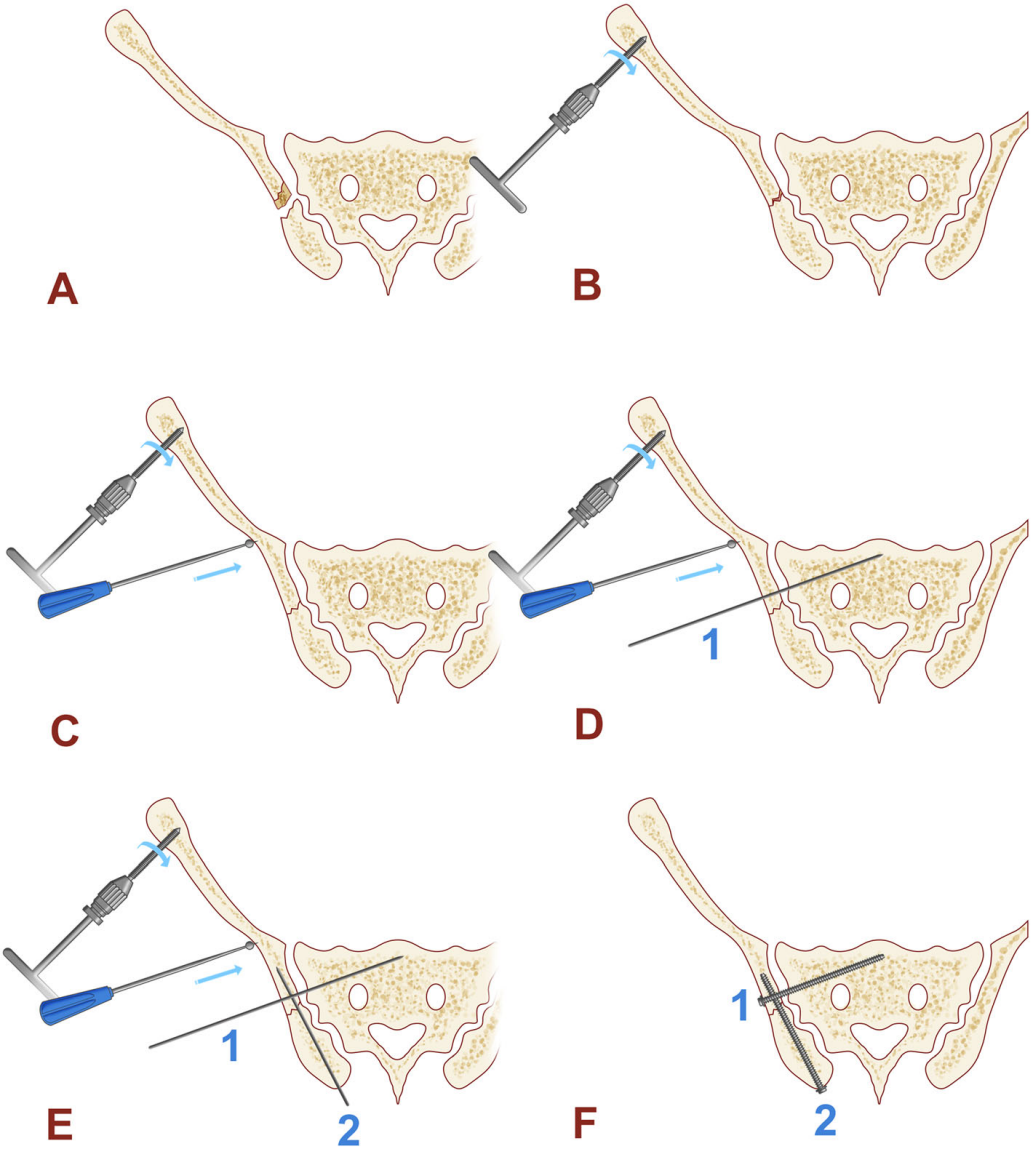
A diagram for the process of fracture reduction and fixation. **a** Pelvic Day type II crescent fracture-dislocation. **b** A 5-mm Schanz nail was inserted into the ilium to correct the rotational displacement. **c** A the top rod was used to correct the separation displacement. **d** Percutaneous insertion of guide pin to fix the sacroiliac joint. **e** Another guide pin was inserted 15° outwardly in the transverse plane and 30° downwardly in the sagittal plane to fix the iliac fracture. **f** Screwed the hollow compression screws with a diameter of 6.5 mm or 7.3 mm along the guide pin

**Fig. 2:**
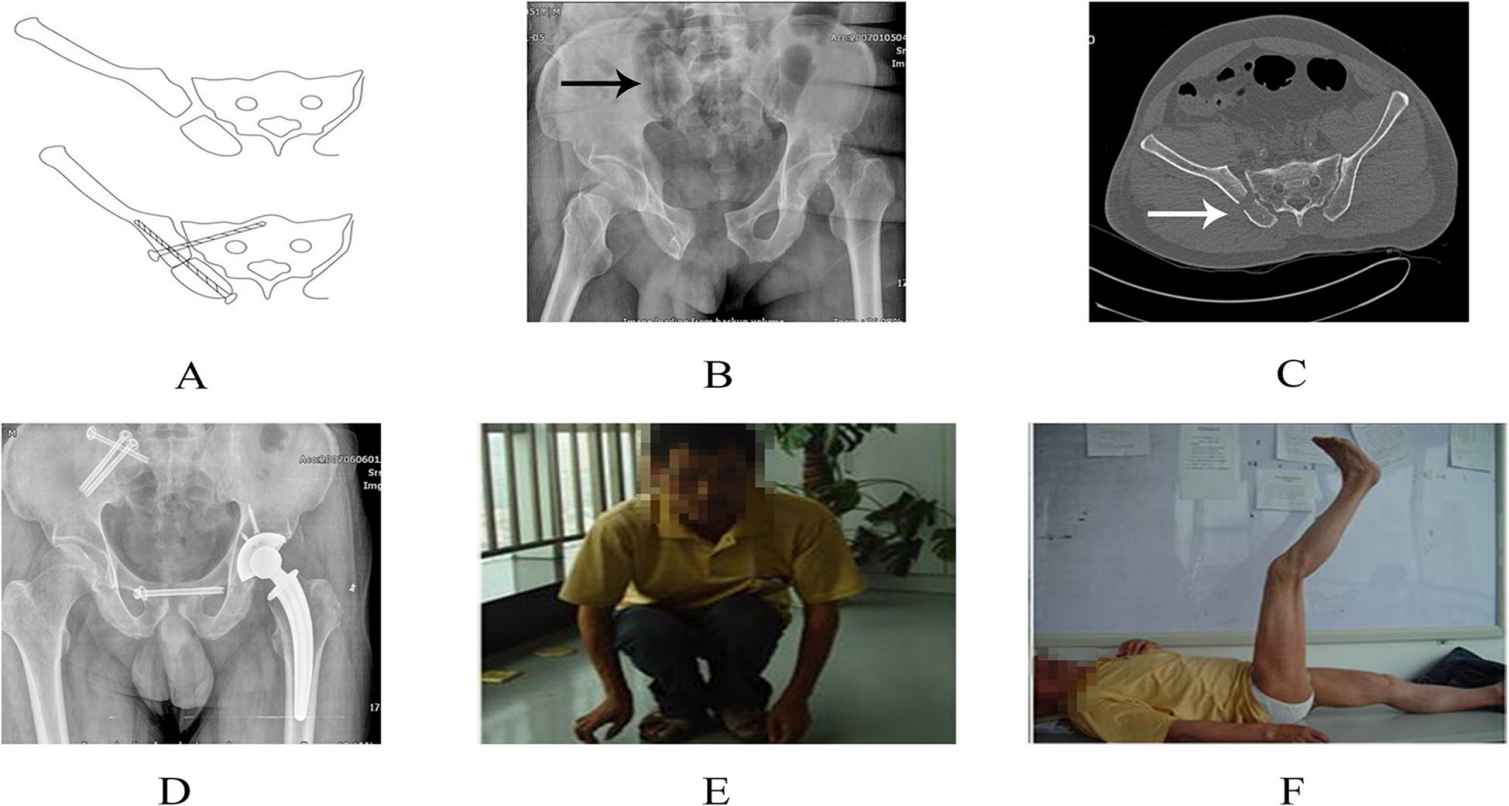
The typical Day type-II crescent fracture-dislocation case. **a** Preoperative schematic diagram. **b** Preoperative X-ray. **c** Preoperative CT examination. **d** Postoperative X-ray. **e**\**f** Postoperative functional evaluation

After the fracture was reduced, the insertion point of the posterior iliac screw on the central side of the posterior superior iliac spine was identified with the aid of fluoroscopy. The guide pin pointed to the anterior inferior iliac spine, and inserted 15° outwardly in the transverse plane and 30° downwardly in the sagittal plane. The lateral pelvic image and the obturator oblique position image should be repeatedly seen to ensure that the guide pin was located above the large ischial notch and in the center of the tear drop. Finally, the depth of the guide pin was measured by the ilium oblique position image. If necessary, the second guide pin could be inserted 1 to 2 cm above and outside from the first one. Screwed the hollow compression screw with a diameter of 6.5 mm or 7.3 mm along the guide pin, and made sure that the screw was in the iliac bone (the operation diagram was shown in Fig. 1 and typical case was shown in Fig. 2).

#### ORIF (group B)

In 10 cases, reconstruction plates and screws were placed to fix the sacroiliac joint and the iliac fracture through the anterior ilioinguinal approach. The remaining 16 patients were treated with posterior approach. Plate and screws were used to fix the iliac fracture, and hollow screws were used to fix the sacroiliac joint. Drainage tubes were routinely placed in this group.

### Postoperative treatment

All patients underwent pelvic radiograph examination on the second day after surgery. The drainage tube was removed within 24 to 72 h after the operation depending on the drainage fluid. The use of antibiotics was according to the incision level. Anticoagulation therapy was required after surgery unless the patients had contraindications. Encouraged patients to perform roll-over exercises in bed 1 day after surgery. Partial weight-bearing exercise was performed with double crutches 3 months after the operation, and full weight-bearing walking was allowed 4 to 6 months postoperatively.

### Follow-up and outcome evaluation

All patients were followed up at 6 weeks, 3, 6, and 12 months after surgery, and then followed up every year. Pelvic X-ray examination, Majeed score, physical examination, and neurological function examination were performed at each follow-up. Radiological results were graded according to the maximum residual displacement of the posterior or anterior pelvic ring injury (excellent, < 4 mm; good, 5-10 mm; fair, 11-20 mm; poor, > 20 mm) [14]. Functional outcomes were measured at the last follow-up according to the criteria described by Majeed et al. [15], which is based on pain, sitting, walking, sexual intercourse, and work (excellent, 85-100 points; good, 70-84 points; fair, 55-69 points; poor, < 55 points).

### Statistical analysis

Statistical analysis was performed using SPSS 18.0 (SPSS Inc., Chicago, Ill). Continuous variables were expressed as mean ± standard deviation. Comparisons between groups were performed using the Student’s *t* test, chi-square test, Fisher’s exact test, and Wilcoxon rank-sum test. *P* value less than 0.05 was considered statistically significant.

## Results

There were no significant differences between the two groups with regard to age, sex, injury mechanisms, and fracture classification(*P* > 0.05, Table 1). Perioperative data were compared between the two groups in Table 2. The average time from injury to surgery was 3.5 ± 2.0 days in group A and 4.0 ± 2.2 days in group B (*P* = 0.365). The mean operative time was significantly less in group A than in group B (36.2 ± 8.8 vs 80.2 ± 9. 1 min; *P* < 0.01). Mean intraoperative blood loss in group A (18.3 ± 8.8 mL) was much less than that in group B (325 ± 78.9 mL, *P* < 0.01). The average incision length in group A was 1.8 ± 0.8 cm, which was significantly longer in the control group (13.2 ± 2.0 cm, *P* < 0.01). There was a statistically significant difference in hospital stay between group A and group B, 4.3 ± 1.6 days and 9.2 ± 4.5 days, respectively (*P* < 0.01).

**Table 1:** General characteristics and fracture data

Variable	Group A	Group B	*P* value
	*N* = 40	*N* = 26	
Age (years)	36.2 ± 7.7	35.4 ± 7.4	0.429
Sex			0.575
Male	25	18	
Female	15	8	
Injury mechanism			0.941
Traffic accident	22	16	
Falling accident	8	4	
Heavy objects	6	4	
Other causes	4	2	
Tile type			0.630
B2.2	27	19	
B2.3	13	7

**Table 2:** Perioperative data

Variable	Group A	Group B	*P* value
Time to surgery (days)	3.5 ± 2.0	4.0 ± 2.2	0.365
Operative time (min)	36.2 ± 8.8	80.2 ± 9. 1	< 0.01
Blood loss (ml)	18.3 ± 8.8	325 ± 78.9	< 0.01
Incision length (cm)	1.8 ± 0.8	13.2 ± 2.0	< 0.01
Hospital stay (days)	4.3 ± 1.6	9.2 ± 4.5	< 0.01

The reduction quality of the two groups was equal (*P* = 0.784, Table 3). The radiological results in group A were excellent in 23 patients, good in 14 patients, fair in 3 patients, and none had poor results at the last follow-up. In comparison, the results in group B were excellent in 17 patients, good in 7 patients, fair in 2 patients, and none had poor results. The average follow-up time was 13 ± 3.5 months (10-24 months) in group A; and 14 ± 4.0 months (9-26 months) in group B. No significant difference was found in the Majeed score at the last follow-up postoperatively between two groups (*P* = 0.305, Table 4). The Majeed scores in group A were excellent in 22 patients, good in 12 patients, fair in 5 patients, and poor in 1 patient. While 8 patients were assessed as excellent, 10 as good, 6 as fair, and 2 as poor in group B.

**Table 3:** Postoperative radiological results

Group	No. of displacements remaining after reduction				
	Excellent (0-4 mm)	Good (5-10 mm)	Fair (11-20 mm)	Poor (> 20 mm)	*P* value
A (*N* = 40)	23	14	3	0	0.784
B (*N* = 26)	17	7	2	0

**Table 4:** Functional results at the last follow-up

Group	No. of displacements remaining after reduction				
	Excellent (> 85)	Good (70-84)	Fair (55-69)	Poor (< 55)	*P* value
A (*N* = 40)	22	12	5	1	0.225
B (*N* = 26)	8	10	6	2	

Intraoperative complications occurred in three patients in group B (1 massive vascular damage bleeding and 3 iatrogenic neurologic injuries), but none in group A. During the postoperative follow-up, 3 cases of recurrent pain, 3 cases of mild claudication, and 3 case of low back pain were found in group A. In group B, there were 3 cases of recurrent pain, 1 case of mild claudication, and 1 case of sexual dysfunction were found.

## Discussion

Borrelli et al. firstly reported the cases of pelvic crescent fracture-dislocation, and all patients were fixed with hollow screws and plates through posterolateral approach [6, 7]. For the treatment of type II CFD, the main surgical method is ORIF by anterior or posterior approach. The advantages of anterior approach surgery are completely expose, accurately anatomical reduction, and immediately stabilize the sacroiliac joint, but the disadvantages are major surgical incision, long operation time, severe soft tissue damage, and L5 and S1 nerve root injury [16–18]. However, the posterior approach surgery has superiority in exposing the posterior structure of the iliac bone, reducing blood vessel and nerve injury, enough space for placing plate and anatomical reduction of fractures besides the reduction of sacroiliac joint [6–8, 11, 19]. ORIF will cause more intraoperative bleeding, greater surgical trauma, and a relatively higher incidence of postoperative incision complications, which also affects the efficacy of surgery.

In recent years, minimally invasive techniques for closed reduction and percutaneous fixation of pelvic fractures have made great progress [20–23]. On the basis of the Day classification, we tried to apply percutaneous sacroiliac joint screws plus posterior iliac screws to treat type II CFD, achieving the purpose of minimally invasive treatment [24]. In this study, we found that PCSIF has less surgical trauma, less intraoperative blood loss, shorter operation time, and lower infection rate than traditional methods. But the shortcomings were that the quality of sacroiliac joint reduction was unguaranteed and the placement of screws required frequent X-ray confirmation. There was no significant difference between the two groups in terms of postoperative Matta score. The mean Majeed score at the last follow-up in group A was higher than that in group B. However, the difference was not statistically significant and might be related to the small number of patients.

The key to the treatment of type II CFD with percutaneous cross screw internal fixation is reduction, so it is necessary to select the appropriate case. The patients with crescent fracture rupture, abnormal sacral anatomy, and dissatisfaction with closed reduction are not suitable for this operation. In the order of fixation, the sacroiliac joint is fixed first, and then the percutaneous posterior iliac screw is used to fix the iliac fracture. The fluoroscopic examination should be repeated to ensure the guide pin inserts correctly. Piston-like movements should be done during the insertion of the guide pin to feel the guide pin walking in the iliac bone. The more mistakes in placing the guide pin, the more likely the screw will loosen and shift.

There were several limitations in this study. First, this was a retrospective study, and selection bias was unavoidable. Second, the data in this article came from a single center and the amount of data was limited. Third, the follow-up time was relatively short. Furthermore, there was no comparison of anterior approach and posterior approach surgery. It is necessary to expand the sample size in future studies for further comparison. Therefore, a further investigation with a larger sample size and a prospective randomized controlled design is needed.

## Conclusion

Percutaneous cross screw internal fixation for Day II type pelvic crescent fracture-dislocation is safe and effective, but the indications must be strictly controlled. Intraoperative closed reduction technique is still the key. The surgeon needs to have a wealth of experience in open reduction and internal fixation surgery, as well as a certain learning curve.

## Data Availability

Patient data comes from our hospital’s medical records follow-up database, transparent and available.

## Abbreviations

PCSIF: Percutaneous cross screws internal fixation
CFD: Crescent fracture-dislocation
ORIF: Open reduction and internal fixation
